# Whole-genome sequencing-based characterization of *Streptomyces* sp. 6(4): focus on natural product

**DOI:** 10.1099/acmi.0.000466.v3

**Published:** 2023-03-02

**Authors:** Marcela Proença Borba, João Paulo Witusk, Débora Marchesan Cunha, Daiana de Lima-Morales, Andreza Francisco Martins, Sueli Van Der Sand

**Affiliations:** ^1^​ Programa de Pós-graduação em Microbiologia Agrícola e do Ambiente, Instituto de Ciências Básicas da Saúde, Universidade Federal do Rio Grande do Sul, Porto Alegre, Brazil; ^2^​ Núcleo de Bioinformática do Hospital de Clínicas de Porto Alegre, Porto Alegre, Rio Grande do Sul, Brazil; ^3^​ Laboratório de Pesquisa em Resistência Bacteriana (LABRESIS), Hospital de Clínicas de Porto Alegre, Porto Alegre, Rio Grande do Sul, Brazil

**Keywords:** Actinomycetes, biosynthetic gene cluster, genome mining, phytopathogenic fungi, secondary metabolites

## Abstract

We have sequenced the whole genome of *Streptomyces* sp. 6(4) isolated from tomato roots that presents antifungal activity against phytopathogenic fungi, mainly *Bipolaris sorokiniana*. The genome has almost 7 Mb and 3368 hypothetical proteins that were analysed and characterized in Uniprot with the emphasis on biological compounds. Multilocus sequence typing (MLST) analyses were performed in an effort to characterize and identify this isolate, resulting in a new sequence type (ST), classified as ST64. Phenetic and phylogenetic trees were constructed to investigate *Streptomyces* sp. 6(4) evolution and sequence similarity, and the isolate is a strain closer to *Streptomyces prasinus* and *Streptomyces viridosporus *. It is known that the genus *Streptomyces* possess huge metabolic capacity with the presence of cryptic genes. These genes are usually present in clusters, which are responsible for the production of diverse natural products, mainly antibiotics. In addition, 6(4) showed 11 biosynthetic gene clusters through antiSMASH, including 3 polyketide synthase (PKS) and non-ribosomal peptide synthase (NRPS) type clusters.

## Data Summary

This Whole Genome Shotgun project has been deposited at DDBJ/ENA/GenBank under the accession VIFW00000000. Due to the large number of nucleotide sequences, the database accession numbers are found in tables throughout the paper and in the supplementary material.

## Introduction

The genus *Streptomyces* comprises Gram-positive bacteria that are commonly found in the soil and produce a great number of secondary metabolites, many of which have distinct biological activities. These natural products are well known for their antimicrobial activities, and *Streptomyces* compounds comprise 70 % of the antibiotics used in medicine [1, 2]. The discovery of new antibiotics from bacterial sources has been limited to the reisolation of the same molecules by traditional methods [3]. Fortunately, the genome sequencing of Actinomycetes lineages since Bentley *et al.* [4] has demonstrated biosynthetic genes that can be silenced in certain situations but are capable of producing bioactive metabolites [5]. This metabolic richness defined a new basis for natural product research, based on genome sequencing and mining [6, 7], demonstrating that this genus is still a promising source of new compounds.

The genus *Streptomyces* has a problematic history with respect to species identification, where strains with different phenotypes, morphology and biochemistry have identical 16S rRNA sequences [8]. DNA hybridization and DNA fingerprinting were used in phylogenetics studies, but no one has found suitable resolution and reproducibility for identification worldwide [9]. Whole-genome sequencing seems to be the best available technique to identify species in the genus *Streptomyces *. Here, we sequenced the whole genome of *Streptomyces* sp. 6(4), an isolate from our research group that produces antifungal compounds and has been studied for a long time without proper species definition.

## Methods

### Data collection

*Streptomyces* sp. 6(4) was previously isolated from tomato plant root (*Lycopersicon esculentum* from 29°55′8″S, 51°10′41″'W) and has been partially identified morphologcally and biochemically [10]. This isolate produces compounds against Gram-negative and Gram-positive bacteria, besides yeasts and especially filamentous fungi [11]. The DNA was obtained by phenol–chloroform extraction and the strain was stocked in agar discs in 20 % glycerol at −20 °C.

### Whole-genome sequencing (WGS)

The MiSeq platform (Illumina, San Diego, CA, USA) was used for WGS of the streptomycetes isolate. The paired-end library was performed with the Nextera XT DNA Library Prep kit (Illumina). The run was performed with the MiSeq Reagent v2 kit (500 cycles) with a calculated coverage of 100×. *De novo* assembly of the genome was performed using SPAdes (v3.6.2; http://cab.spbu.ru/software/spades/). The file generated by the assembly was annotated on the RAST platform (http://rast.nmpdr.org) and Prokka (contigs ≥200 bp; ≥10× coverage) [12]. The *Streptomyces* sp*.* 6(4) genome was visualized in Geneious Prime 9.0.5. The hypothetical protein annotation was performed after blastx (National Center for Biotechnology Information, NCBI) searches and UniProt blast analysis [13]. antiSMASH 4.0 software [14] was used to predict biosynthetic gene clusters for secondary metabolites.

The six alleles, 16S rRNA, *trpB*, *recA*, *rpoB*, *gyrB* and *atpD*, were available *in silico* and the sequence type (ST) assignments were defined using the *Streptomyces* database at PubMLST [15].

### Phylogenetic and phenetic trees

Multilocus sequence typing (MLST)-based phylogenetic and phenetic trees were developed by aligning concatenated sequences of six conserved genes (16S rRNA, *rpoB*, *trpB*, *recA*, *gyrB* and *atpD*). Gene alignment was performed in Geneious Prime 9.0.5 using ClustalW. Two different algorithms and evolutionary models were used, neighbour joining with the Kimura two-parameter model and maximum likelihood with the general time-reversible model, both with 500 bootstrap replicates with 102 no-trimmed sequences from *Streptomyces* representative genome strains from the NCBI using *Mycobacterium tuberculosis* H37Rv as an external group in megax software [16]. For brevity, non-target clades and groups were coalesced in the figures to highlight the position of 6(4) in both analyses; complete trees are available in Figs S2 and S3 (available in the online version of this article).

A new *Streptomyces* genome related to *S. prasinus* and *S. viridosporus* was deposited in GenBank and the *Streptomyces* sp. 6(4) is able to generate natural products according to its biosynthetic gene clusters.

## Results and discussion

This Whole Genome Shotgun project has been deposited at DDBJ/ENA/GenBank under the accession VIFW00000000. The complete linear genome was 6 868 166 bp with 72.7 % GC content (2477 contigs; N50=7987). Six thousand and thirty-eight protein-coding genes and 76 RNA genes were detected. All 3368 hypothetical proteins were analysed and only 1132 hypothetical proteins had their function identified in Uniprot. Protein analysis in blastx showed that 60 hypothetical proteins are involved in the metabolism process and 72 are involved in biosynthetic metabolism according to Uniprot (Table S1).

With this in view, we identified some proteins in 6(4) that are involved in biosynthetic metabolism and could be investigated in further studies. One of these is a 4,5-DOPA dioxygenase estradiol that is part of the construction process of various biological agents, such as anthramycin, porothramycin, sibiromycin, tomaymycin, hormaomycins and lincomycin A [17]. An antibiotic biosynthesis monooxygenase protein was recognized in 6(4) and seems to be part of some already known pathways of antifungal and antibiotic synthesis, such as nystatin in *Streptomyces noursei* [18] and actinorhodin in *Streptomyces coelicolor* [19], respectively. The thiosterase is another protein that has been identified and it is part of polyketide synthase (PKS) construction [20, 21]. The PKSs and non-ribosomal peptide synthases (NRPSs) are enzymatic complexes that are responsible for the formation of polyketides and non-ribosomal peptides that form two large and important groups of natural products with different chemical structures responsible for a wide variety of biologically active compounds [22]. Another important enzyme involved in the construction of PKSs and NRPSs is the aminotransferase class I/II-fold pyridoxal phosphate-dependent enzyme [23], present in the 6(4) isolate. We also identified a thiazolylpeptide-type bacteriocin. Thiazolyl peptides are antibiotics originated by translation modifications from ribosomal natural products and are found in streptomycetes [24], and thiazole moiety is used in all penicillin derivatives such as thiabendazole, a known fungicide [25]. Terpenoids are a diverse group of natural products with antifungal properties [26], commonly found in plants, and need two initial primary metabolites to be produced, isopentenyl diphosphate (IPP) and dimethylallyl diphosphate (DMAPP). Both carbon units are originated by distinct pathways. The 3-hydroxy-3-methylglutaryl-ACP synthase, observed in 6(4), is present in the genus *Streptomyces* and is responsible for terpenoid biosynthesis by the mevalonate pathway [27]. Squalene synthase HpnD is also a protein required by the mevalonate pathway to produce squalene, a kind of terpenoid from the isoprenoid group [28]. Another independent pathway for terpenoids involves 1-deoxy-d-xylulose-5-phosphate synthase, which condenses pyruvate and glyceraldehyde 3-phosphate [29, 30]. Both pathways seem to be present in 6(4). Anthranilate phosphoribosyltransferase is involved in the biosynthesis of a nonribosomal lipopeptide called a calcium-dependent antibiotic (CDA) in, at least, *S. coelicolor* [31], as with nucleotide sugar dehydrogenase that is the precursor of congocidine biosynthesis [32]. Both enzymes were found in this 6(4)isolate. Other proteins were identified in this study as biosynthesis proteins. Many of them were part of carbohydrate metabolism, acid folic metabolism, amino acids and vitamins. In these cases, nothing was uncovered about biological compounds, but this does not mean that these enzymes do not participate in natural product production; we simply do not know yet, and it is very common for these metabolism pathways to generate active compounds by some means. Further, some proteins described above seems to be part of diverse known antifungal products, which can corroborate the antifungal action of 6(4) against *B. sorokiniana*.

The first streptomycete that has had its whole genome sequenced – because of important bioactive compounds derived from secondary metabolites [4] – belongs to *S. coelicolor *. The authors have sequenced the genome of *S. coelicolor* A3(2) and discovered just by analysing the genome that, besides the 5 compounds already known, the isolate could produce approximately 20 different compounds. Bentley’s study showed that this group of bacteria is still a source for the discovery of new metabolites with biological activities. This and other studies [33, 34] showed the existence of cryptic genes that are silent in certain conditions and are the missing gap in the search for new natural products. With the whole genome sequence, genome mining is used to find the biosynthetic gene clusters (BGCs) in *Streptomyces *, known for the large number of these clusters in their genomes. These BGCs are categorized into 34 major classes [35]. In antiSMASH we identified 11 gene clusters encoding pathways for secondary metabolism. All proteins have been searched in Geneious software and blastp to confirm the mining (Table 1).

Two clusters are terpene types. The terpenoids are famous natural products and can be produced by the mevalonate pathway, as seen above. These terpenoids are widely produced by *Streptomyces* [36] and this strain can probably produce them too. The BGC in the 186.1 region, found in 6(4), showed sequence similarity with terpene BGCs in *Streptomyces griseus* subsp. *griseus* NBRC 13350 (BGC000664 [37]), *Streptomyces griseus* (BGC0000649 [38]), *Streptomyces collinus* (BGC0001227 [39]), *Streptomyces scabiei* (BGC0001456 [40]) and *Streptomyces avermitilis* (BGC0000633 [41]) (Fig. S1). Aryl polyene in region 29.1 is a pigment polyketide originated by the PKS system [42] and at least another three clusters encode PKS and NRPS genes, an important discovery indicating that 6(4) has distinct antimicrobial activity, e.g. antifungal, acting against a variety of micro-organisms, as shown in later studies [11].

AntiSMASH analysis confirmed that this strain has different gene clusters and can produce different biological compounds. Although it is very common to find hopene in the *Streptomyces* genome because of aerial growth of the mycelia, in 6(4) it was not possible to detect it. This is not a problem due to the fact that not all streptomycetes necessarily seem to produce this compound [43]. However, we have not been able to identify geosmin and this could be due to some mishap in the sequencing process.

**Table 1.** Clusters and proteins identified in *Streptomyces* 6(4) analysis in antiSMASH

Region	Type	Proteins
4.1	Melanin	Tyrosinase
		Cytochrome P450
29.1	Arylpolyene	Hypothetical protein
		3-oxoacyl-ACP synthase
		o-succunylbenzoate--CoA ligase
100.1	Terpene	Sporulation protein
		Hypothetical protein
		Lycopene cyclase
140.1	RiPP-like	Endonuclease
165.1	PKS-like	Endonuclease
		Type I polyketide synthase
		Acyl-carrier-protein S-malonyltransferase
186.1	Terpene	Phytoene synthase
		2Fe-2S ferredoxin
207.1	RiPP-like	Hypothetical protein
		Endonuclease
243.1	NRPS-like	Serine hydroxymethyltransferase
		Non-ribosomal peptide synthetase
268.1	T1PKS	Type I polyketide synthase
276.1	NRPS	Non-ribosomal peptide synthetase
327.1	NRPS	Non-ribosomal peptide synthetase

The genome was analysed in PubMLST and classified as ST64, a new ST deposited in the website as id 271.

**Fig. 1.**
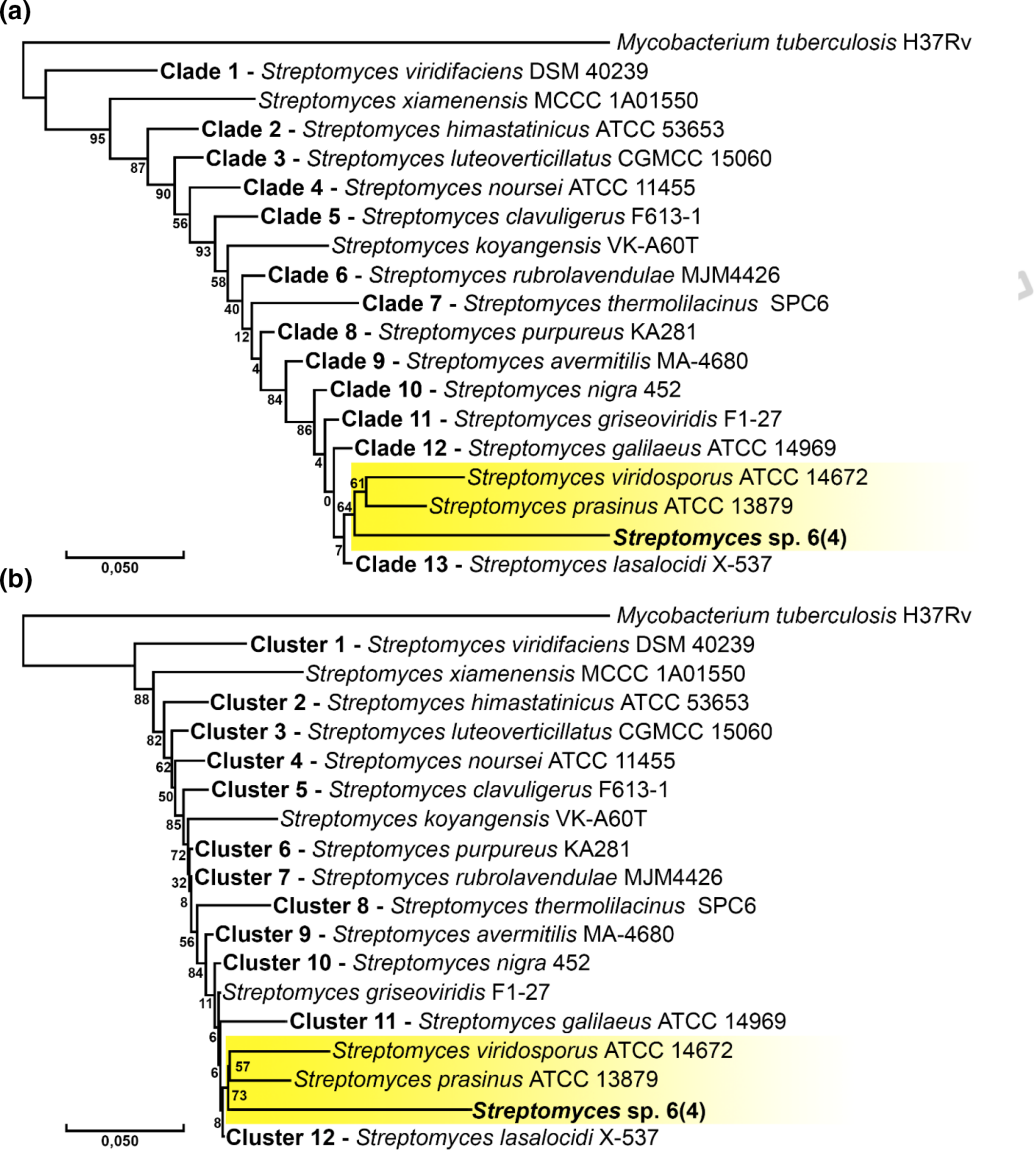
Maximum-likelihood phylogenetic tree with the general time-reversible model (a) and neighbour-joining phenetic tree with the Kimura two-parameter model (b) for *Streptomyces* sp. 6(4) with 102 *Streptomyces* representative genome strains coalesced into clusters for a clearer view (see Figs S2 and S3). *M. tuberculosis* H37Rv is used as an external group (Table S3).

A phylogenetic tree was constructed to infer evolutionary relationships with the maximum-likelihood algorithm (Fig. 1a) and a phenetic tree was constructed to observe sequence similarities, using neighbour joining (Fig. 1b). Both trees aim to characterize the 6(4) and are commonly used to deduce species identification in microbiology. In this study, the *Streptomyces* sp. 6(4) is related to *S. prasinus* ATCC 13879 and *S. viridosporus* ATCC 14672, forming a sister group to these taxa in both attempted trees (Fig. 1), showing high similarity and a close relationship with these two species.

Morphological description and MLSA analysis of *S. prasinus* were presented by Labeda *et al.* [44] and it was found that *S. prasinus* shared morphological traits with *S. viridosporus* according to Goodfellow *et al.* [45]. Although the 16S rRNA showed high similarity with *Streptomyces griseoincarnatus* when analysed in EzBioCloud [46], in the NCBI database it showed similarity with *Streptomyces griseoflavus.* The MLST genes were investigated in the NCBI database, but lower percentage rates were achieved. Some species (*Streptomyces cadmiisoli, S. collinus, Streptomyces hygroscopicus, S. incarnatus* and *Streptomyces olivaceus *) appear in all housekeeping genes. *S. coelicolor, Streptomyces albogriseolus* and *Streptomyces ambofaciens* are species that also appear in all housekeeping genes and in the 16S rRNA, but with different similarity percentages (Table S2). Even with the complete genome sequenced, we cannot define the species that fits this isolate genomically, as usual with members of the genus *Streptomyces *, due to the large number of species and poor variation in 16S rRNA [47]. The publication of new species belonging to the genus requires several methods in order to reach a final conclusion [48], since the methods separately are not sufficient even for the exact identification of already known species. This is due to the large number of species attributed to the genus – approximately 3000 different species – especially in patent records [49]. Today, searching for *Streptomyces* at www.bacterio.net, we arrived at 696 species in validated publications and corrected names without synonyms, which is still a large number of species belonging to the same genus, making the taxonomy of streptomycetes more complicated than in other bacterial genera.

This paper also aims to show the variety of metabolic possibilities in the genome of one *Streptomyces* isolate but we cannot affirm that any biosynthetic gene cluster present in 6(4) is activated just because the isolate has activity against phytopathogenic fungi.

## Supplementary Data

Supplementary material 1
