# Supplementary material for: Whole-genome sequencing-based characterization of Streptomyces sp. 6(4): focus on natural product
Source: Access Microbiol. 2023 Mar 2;5(3):acmi000466.v3. doi: 10.1099/acmi.0.000466.v3 (PMC10118248; doi:10.1099/acmi.0.000466.v3)
Supplement: Supplementary material 1 [file acmi-5-466.v3-s001.pdf]

Supplementary Material

Whole Genome Sequencing Based Characterization of *Streptomyces* sp. 6(4): focus on natural products

Marcela Proença Borba[1], João Paulo Witusk[1], Débora Marchesan Cunha[1], Daiana de Lima-Morales[2,3], Andreza Francisco Martins[1,2], Sueli Van Der Sand[1]

1- Programa de Pós-graduação em Microbiologia Agrícola e do Ambiente, Instituto de Ciências Básicas da Saúde, Universidade Federal do Rio Grande do Sul, Porto Alegre, Brazil
2- Núcleo de Bioinformática do Hospital de Clínicas de Porto Alegre, Rio Grande do Sul, Porto Alegre, Brazil
3- Laboratório de Pesquisa em Resistência Bacteriana (LABRESIS), Hospital de Clínicas de Porto Alegre, Rio Grande do Sul, Porto Alegre, Brazil

Table S1: Protein groups present in *Streptomyces* 6(4) according Uniprot (% refers to 1,132 hypothetical proteins with function identified).

Metabolic process	Nº	%	Biosynthetic Process	Nº	%
Alkaloid	2	0,18	Acetyl-CoA	2	0,18
Antibiotic	1	0,9	Amino acids	4	0,35
Aromatic amino acids	3	0,26	Asparagine	2	0,18
Carbohydrate	18	1,6	Coenzyme A	1	0,9
Cell wall macromolecule	1	0,9	Cyclic nucleotide	1	0,9
Cellular amino acids	6	0,53	dTPD	2	0,18
Cellular aromatic compound	1	0,9	Ergosterol	1	0,9
Cysteine	1	0,9	Fatty acids	3	0,26
DNA	1	0,9	Folic acid	3	0,26
Fatty acid	2	0,18	Glutamate	1	0,9
Fructose 6-phosphate	1	0,9	Glutamine	1	0,9
Glucose	1	0,9	Glycogen	2	0,18
Glutamine	1	0,9	Histidine	1	0,9
Glyoxylate	1	0,9	Inosine monophosphate	2	0,18
Guanosine tetraphosphate	1	0,9	Isoprenoid	1	0,9
L-arabinose	1	0,9	Lysine via diaminopimelate	2	0,18
Lipid	3	0,26	Melanin	1	0,9
Nucleoside	1	0,9	Menaquinone	1	0,9
Protein	2	0,18	Methionine	2	0,18
Protein phosphorylation (secondary metabolites)	2	0,18	NAD	1	0,9
			Nucleoside	1	0,9
Pteride-containing compound	3	0,26	Peptidoglycan	2	0,18
Pyrimide nucleoside	2	0,18	Phospholipid	2	0,18
Pyruvate	3	0,26	Photoporphyrinogen	2	0,18
Unknow	2	0,18	Polysaccharide	4	0,35
			Riboflavin	8	0,7
			Spermidine	1	0,9
			Steroid	1	0,9
			Terpenoid	3	0,26
			Tetrapyrrole	1	0,9
			Tryptophan	1	0,9
			Unknow	12	1,1

Table S2: Species with sequence similarity to *Streptomyces* sp. 6(4) analyzed in GenBank/NCBI. Only sequences with published research papers were accepted.

Gene	Species	Acession Number	Query (%)	Id (%)
16S rRNA	*S. griseoflavus*	EU741217.1	98	99,60
	S. coelicolor	HQ848084.1	99	99,34

	S. variabilis	NR_043840.1	98	99,33
	S. aureofaciens	AY289116.1	98	99,14
	S. albogriseolus	CP042594.1	100	98,89
	S. althioticus	NR_115392.1	98	98,87
	S. ambofaciens	CP012949.1	100	98,82
	S. ambofaciens	AM238663.1	100	98,82
	S. ambofaciens	M27245.1	100	98,82
	S. albogriseolus	AJ494865.1	99	98,82
	S. pseudogriseolus	X80827.1	99	98,75
	S. tendae	NR_025871.2	100	98,69
	S. tendae	D63873.1	100	98,69
atpD	*S. albogriseolus*	CP042594.1	100	96,24
	S. ambofaciens	CP012949.1	98	96,03
	S. coelicolor	CP042324.1	100	95,89
	S. cadmiisoli	CP030073.1	100	95,89
	S. coelicolor	AL939123.1	100	95,89
	S. lividans	Z22606.1	100	95,89
	S. collinus	CP006259.1	100	95,69
	S. hygroscopicus	CP003275.1	100	95,34
	S. olivaceus	CP016795.1	100	95,27
	S. murinus	CP066774.1	100	95,00
	S. nodosus	CP009313.1	100	94,92
	S. rochei	AP018517.1	100	94,86
	S. fodineus	CP017248.1	100	94,78
	S. cyaneochromogenes	CP034539.1	100	94,29
	S. davawensis	HE971709.1	100	94,22
	S. incarnatus	CP011497.1	100	94,02
	S. avermitilis	BA000030.4	100	93,95
	S. scabiei	FN554889.1	100	93,67
	S. spectabilis	CP040916.1	100	93,47
gyrB	*S. albogriseolus*	CP042594.1	100	90,65
	S. cadmiisoli	CP030073.1	100	90,41
	S. coelicolor	CP042324.1	99	90,39
	S. coelicolor	AL939125.1	99	90,39
	S. cyaneochromogenes	CP034539.1	100	90,31
	S. ambofaciens	CP012949.1	99	90,29
	S. collinus	CP006259.1	100	90,12
	S. davawensis	HE971709.1	100	90,07
	S. tuirus	AP023439.1	100	90,07
	S. olivaceus	CP016795.1	99	90,01
	S. incarnatus	CP011497.1	100	89,53

	S. hygroscopicus	CP003275.1	100	89,46
	S. nodosus	CP009313.1	100	89,33
	S. fodineus	CP017248.1	100	88,99
	S. lincolnensis	CP022744.1	99	89,02
	S. lincolnensis	CP046024.1	99	89,02
	S. murinus	CP066774.1	99	88,64
	S. scabiei	FN554889.1	100	88,45
recA	*S. albogriseolus*	CP042594.1	100	94,74
	S. laurentii	AP017424.1	92	94,71
	S. fodineus	CP017248.1	100	94,33
	S. cyaneochromogenes	CP034539.1	100	94,05
	S. tuirus	AP023439.1	100	93,81
	S. hygroscopicus	CP003275.1	100	93,71
	S. olivaceus	CP016795.1	100	93,62
	S. cadmiisoli	CP030073.1	100	93,39
	S. collinus	CP006259.1	100	93,35
	S. davawensis	HE971709.1	100	93,33
	S. incarnatus	CP011497.1	100	93,26
	S. lividans	X76076.1	100	93,26
	S. coelicolor	CP042324.1	100	93,17
	S. coelicolor	AL939125.1	100	93,17
	S. ambofaciens	CP012949.1	100	93,00
	S. rochei	AP018517.1	100	92,91
	S. avermitilis	BA000030.4	99	92,49
	S. nodosus	CP009313.1	99	92,48
rpoB	*S. actuosus*	FJ438820.1	100	95,78
	S. albogriseolus	CP042594.1	100	95,47
	S. cadmiisoli	CP030073.1	100	95,44
	S. nodosus	CP009313.1	100	95,41
	S. tuirus	AP023439.1	100	94,89
	S. cyaneochromogenes	CP034539.1	100	94,78
	S. davawensis	HE971709.1	100	94,66
	S. hygroscopicus	CP003275.1	100	94,61
	S. rochei	AP018517.1	100	94,58
	S. fodineus	CP017248.1	100	94,58
	S. collinus	CP006259.1	100	94,52
	S. murinus	CP066774.1	100	94,47
	S. incarnatus	CP011497.1	100	94,38
	S. olivaceus	CP016795.1	100	94,33
	S. coelicolor	CP042324.1	100	94,29
	S. coelicolor	AL939121.1	100	94,29

	S. incarnatus	AB516306.1	100	94,24
	S. ambofaciens	CP012949.1	100	94,21
	S. scabiei	FN554889.1	100	94,04
trpB	*S. albogriseolus*	CP042594.1	90	92,08
	S. laurentii	AP017424.1	84	92,05
	S. murinus	CP066774.1	87	91,36
	S. rochei	AP018517.1	87	91,14
	S. olivaceus	CP016795.1	90	91,11
	S. venezuelae	FR845719.1	85	90,95
	S. lincolnensis	CP022744.1	90	90,70
	S. lincolnensis	CP046024.1	90	90,70
	S. collinus	CP006259.1	99	90,55
	S. coelicolor	CP042324.1	94	90,47
	S. coelicolor	AL939111.1	94	90,47
	S. coelicolor	AF054585.1	94	90,47
	S. hygroscopicus	CP003275.1	91	90,35
	S. ambofaciens	CP012949.1	99	88,57
	S. cadmiisoli	CP030073.1	99	87,62

Table S3: Species list of Representative Genomes from NCBI used to construct both trees.

Species	Strain	Acession Number
S. actuosus	ATCC 25421	NZ_CP029788
S. agglomeratus	6-3-2	NZ_MEHJ01000001
S. albireticuli	MDJK11	NZ_CP021744
S. alboflavus	MDJK44	NZ_CP021748
S. alboniger	ATCC 12461	NZ_CP023695
S. albulus	NR660	NZ_CP007574
S. alfalfae	ACCC 40021	NZ_CP015588
S. ambofaciens	ATCC 23877	NZ_CP012382
S. antimycoticus	NBRC 12839	NZ_BJHV01000001
S. aquilus	GGCR-6	NZ_CP034463
S. asteoporus	DSM 41452	NZ_CP022310
S. aureoverticillatus	HN6	NZ_CP048641
S. autolyticus	CGMCC 0516	NZ_CP019458
S. avermitilis	NBRC 14893	NC_003155
S. bacillaris	ATCC 15855	NZ_CP029378
S. badius	SP6C4	NZ_LWMQ01000001
S. bingchenggensis	BCW-1	NC_016582
S. bottropensis	ATCC 25435	NZ_KB911581
S. brevispora	DSM 42059	NZ_VIWW01000001
S. capillispiralis	DSM 41695	NZ_VIWV01000001

S. chartreusis	ATCC 14922	NZ_CP023689
S. chrestomyceticus	JCM 4735	NZ_BHZC01000001
S. cinereoruber	ATCC 19740	NZ_CP023693
S. clavuligerus	F613-1	NZ_CP016559
S. coelicolor	A3(2)	NC_003888
S. coeruleorubidus	ATCC 13740	NZ_CP013684
S. collinus	Tu365	NC_021985
S. cyaneogriseus subsp. *noncyanogenus*	NMWT	NZ_CP010849
S. davaonensis	JCM 4913	NC_020504
S. dengpaensis	X2HG99	NZ_CP026652
S. exfoliatus	A1013Y	NZ_CP040244
S. ficellus	NRRL 8067	NZ_CP04279
S. filamentous	NRRL 11379	NZ_ABXX02000001
S. fodineus	TW1S	NZ_CP017248
S. formicae	KY5	NZ_CP022685
S. fradiae	ATCC 10745	NZ_CP023696
S. fulvissimus	DSM 40593	NC_021177
S. fungicidicus	TXX3120	NZ_CP023407
S. galilaeus	ATCC 14969	NZ_CP023703
S. gilvosporeus	F609	NZ_CP020569
S. glaucescens	GLA.O	NZ_CP009438
S. globisporus	TFH56	NZ_CO029361
S. globosus	LZH-48	NZ_CP030862
S. griseochromogenes	ATCC 14511	NZ_CP016279
S. griseoflavus	Tu4000	NZ_GG657758
S. griseorubiginosus	3E-1	NZ_CP032427
S. griseoviridis	F1-27	NZ_CP034687
S. griseus subsp. *griseus*	NBRC 13350	NC_010572
S. himastatinicus	ATCC 53653	NZ_GG657754
S. hundungensis	BH38	NZ_CP032698
S. iranensis	SCAF00001	NZ_LK022848
S. kanamyceticus	ATCC 12853	NZ_CP023699
S. kaniharaensis	SF-557	NZ_VDEQ01000305
S. katrae	S3	NZ_CP020042
S. koyangensis	VK-A60T	NZ_CP031742
S. lasalocidi	X-537	NZ_SZNQ01000001
S. lavendulae subsp. *lavendulae*	CCM 3239	NZ_CP024985
S. libani subsp. *libani*	NBRC 13452	NZ_BLIPO1000001
S. lincolnensis	NRRL 2936	NZ_CP016438
S. lunaelactis	MM109	NZ_CP026304
S. luteoverticillatus	CGMCC 15060	NZ_CP034587
S. lydicus	103	NZ_KN050734
S. malaysiensis	DSM 4137	NZ_CP023992

S. melanosporofaciens	DSM 40318	NZ_FNST01000002
S. mexicanus	Q0842	NZ_LR732544
S. misionensis	DSM 40306	NZ_FNTD01000004
S. nigra	452	NZ_CP029043
S. nitrosporeus	ATCC 12769	NZ_CP023702
S. niveus	SCSIO 3406	NZ_CP018047
S. nodosus	ATCC 14889	NZ_CP009313
S. noursei	ATCC 11455	NZ_CP011533
S. pactum	ACT 12	NZ_CP019724
S. parvulus	2297	NZ_CP015866
S. peucetius subsp. *caesius*	ATCC 27952	NZ_CP022438
S. platensis	ATCC 23948	NZ_CP023691
S. pluripotens	MUSC 135	NZ_CP021080
S. prasinus	ATCC 13879	NZ_CP023697
S. pristinaespiralis	HCCB 10218	NZ_CP011340
S. puniscicabiei	DSM 41929	NZ_VFNX01000001
S. purpureus	KA281	NZ_KB913030
S. qaidamensis	S10	NZ_CP015098
S. qinzhouesis	SLL-25	NZ_CP042266
S. roseochromogenes subsp. *oscitans*	DS 12976	NZ_CM002285
S. rubvolavendulae	MJM4426	NZ_CP017316
S. scabiei	87.22	NC_013929
S. seoulensis	KCTC 9819	NZ_CP032229
S. spectabilis	ATCC 27465	NZ_CP023690
S. spongiicola	HNM0071	NZ_CP029254
S. subrutilus	ATCC 27467	NZ_CP023701
S. sviceus	ATCC 29083	NZ_CM000951
S. tendae	139	NZ_CP043959
S. thermolilacinus	SPC6	NZ_ASHX02000001
S. tirandamycinicus	HNM0039	NZ_CP029188
S. tubercidicus	NBRC 13090	NZ_BLIR0100000.1
S. venezuelae	ATCC 10712	NZ_CP029197
S. vietnamensis	GIMV4.0001	NZ_CP010407
S. vinaceus	ATCC 27476	NZ_CP023692
S. violaceoruber	S21	NZ_CP020570
S. violaceusniger	NBRC 13459	NZ_BJHW01000001
S. viridifaciens	DSM 40239	NZ_MPLE01000004
S. viridosporus	ATCC 14672	NZ_DS999641.1
S. xiamenensis	MCCC 1AO1550	NZ_CP009922

Figure S1: Similar BGCs founded in antiSMASH analysis of *Streptomyces* sp. 6(4) cluster region 186.1.

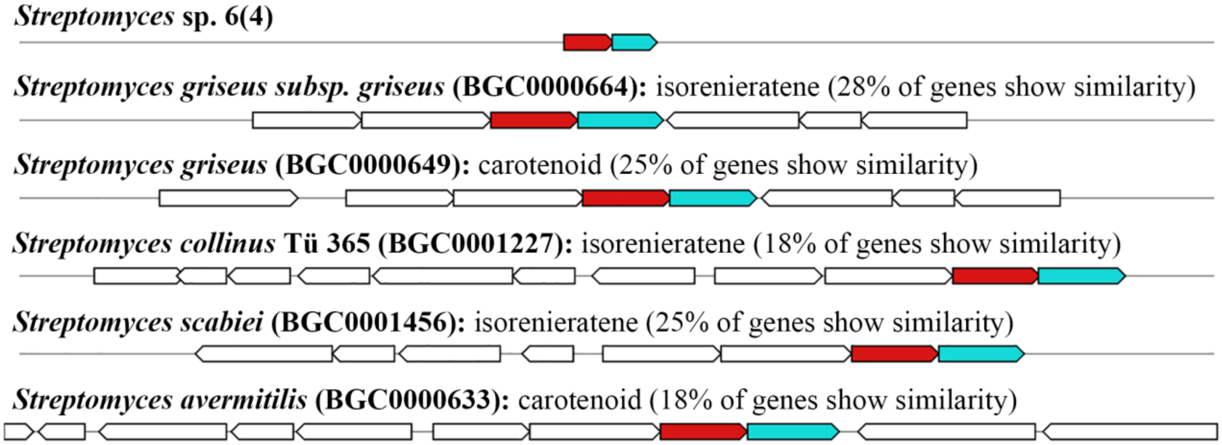

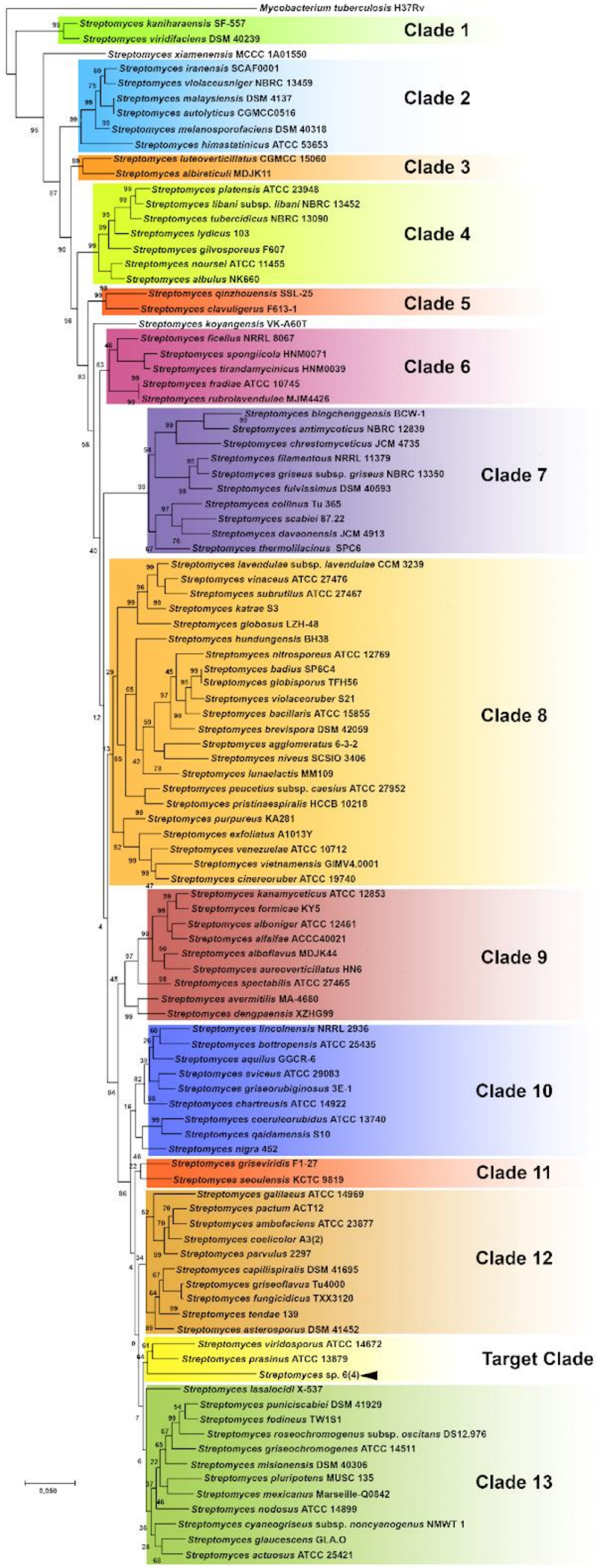

Figure S2: Phylogenetic tree with coalesced clades.

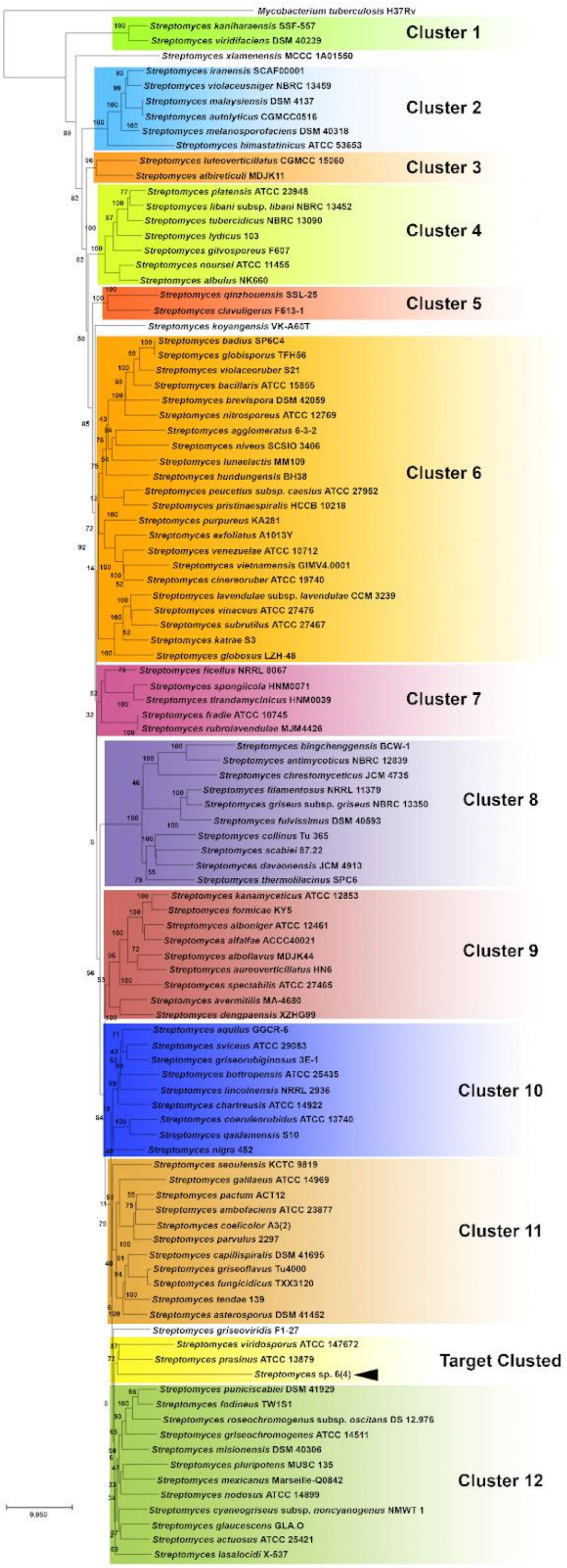

Figure S3: Phenetic tree with coalesced clusters.
